# Pain Care Disparities and the Use of Virtual Care Among Racial-Ethnic Minority Groups During COVID-19

**DOI:** 10.1007/s11606-023-08473-0

**Published:** 2024-01-22

**Authors:** Sarah J. Javier, Evan P. Carey, Elise Gunzburger, Huang-Yuan P. Chen, Steven B. Zeliadt, Emily C. Williams, Jessica A. Chen

**Affiliations:** 1 Center for Innovation to Implementation (Ci2i), VA Palo Alto Healthcare System, 795 Willow Road (152-MPD), Menlo Park, CA 94025 USA; 2 Stanford University School of Medicine, Stanford, CA USA; 3 Center of Innovation for Veteran-Centered and Value-Driven Care, VA Eastern Colorado Health Care System, Denver, CO USA; 4 Department of Biostatistics and Informatics, Colorado School of Public Health, University of Colorado Anschutz Medical Campus, Aurora, CO USA; 5 Center of Innovation for Veteran-Centered and Value-Driven Care, VA Puget Sound Health Care System, Seattle, WA USA; 6 Department of Health Systems and Population Health, School of Public Health, University of Washington, Seattle, WA USA; 7 Department of Psychiatry and Behavioral Science, University of Washington, Seattle, WA USA

**Keywords:** COVID-19, disparity, pain, telehealth, veteran, virtual care.

## Abstract

**Background and Objective:**

COVID-19 led to an unprecedented reliance on virtual modalities to maintain care continuity for patients living with chronic pain. We examined whether there were disparities in virtual specialty pain care for racial-ethnic minority groups during COVID-19.

**Design and Participants:**

This was a retrospective national cohort study with two comparison groups: primary care patients with chronic pain seen immediately prior to COVID-19 (3/1/19–2/29/20) (*N* = 1,649,053) and a cohort of patients seen in the year prior (3/1/18–2/28–19; *n* = 1,536,954).

**Main Measures:**

We assessed use of telehealth (telephone or video) specialty pain care, in-person care specialty pain care, and any specialty pain care for both groups at 6 months following cohort inclusion. We used quasi-Poisson regressions to test associations between patient race and ethnicity and receipt of care.

**Key Results:**

Prior to COVID-19, there were Black-White (RR = 0.64, 95% CI [0.62, 0.67]) and Asian-White (RR = 0.63, 95% CI [0.54, 0.75]) disparities in telehealth use, and these lessened during COVID-19 (Black-White: RR = 0.75, 95% CI [0.73, 0.77], Asian-White: RR = 0.81, 95% CI [0.74, 0.89]) but did not disappear. Individuals identifying as American Indian/Alaska Native used telehealth less than White individuals during early COVID-19 (RR = 0.98, 95% CI [0.85, 1.13] to RR = 0.87, 95% CI [0.79, 0.96]). Hispanic/Latinx individuals were less likely than non-Hispanic/Latinx individuals to use telehealth prior to COVID-19 but more likely during early COVID-19 (RR = 0.70, 95% CI [0.66, 0.75] to RR = 1.06, 95% CI [1.02, 1.09]). Disparities in virtual pain care occurred over the backdrop of overall decreased specialty pain care during the early phase of the pandemic (raw decrease of *n* = 17,481 specialty care encounters overall from pre-COVID to COVID-era), including increased disparities in any VA specialty pain care for Black (RR = 0.81, 95% CI [0.80, 0.83] to RR = 0.79, 95% CI [0.77, 0.80]) and Asian (RR = 0.91, 95% CI [0.86, 0.97] to RR = 0.88, 95% CI [0.82, 0.94]) individuals.

**Conclusions:**

Disparities in virtual specialty pain care were smaller during the early phases of the COVID-19 pandemic than prior to the pandemic but did not disappear entirely, despite the rapid growth in telehealth. Targeted efforts to increase access to specialty pain care need to be concentrated among racial-ethnic minority groups.

**Supplementary Information:**

The online version contains supplementary material available at 10.1007/s11606-023-08473-0.

## BACKGROUND

Chronic pain is a debilitating condition that affects millions of people in the United States.^1^ Chronic pain not only affects individuals somatically, but can exacerbate psychological and social complications when left unmanaged.^2^ If individuals with chronic pain do not have access to timely, continuous, and evidence-based pain care, they are at increased risk for suicide and premature death.^3–6^

In the 2010s, there was a national shift in pain care from opioid prescribing and ineffective, often-invasive procedures to a comprehensive model utilizing non-pharmacological treatments.^7–9^ Likewise, in 2015, the Veterans Health Administration (VA) prioritized access to evidence-based non-pharmacological pain treatments (herein referred to as “specialty pain care”), including complementary and integrative health modalities, psychosocial treatments, physical medicine, and rehabilitation services.^10–12^ As of 2019, 78 out of 102 VA medical facilities had either a multidisciplinary pain center or clinic as their highest-functioning pain care setting, indicating wider access to these treatments.^13^ It is likely that that number has increased since 2019 given a federal mandate for all VA medical facilities to have interdisciplinary pain management teams.^8^

In March 2020, the novel coronavirus-19 pandemic (COVID-19) caused unprecedented disruptions to healthcare delivery, including specialty pain care.^14^ Certain specialty pain care services traditionally delivered in-person (e.g., physical therapy) were greatly reduced in availability.^14^ To mitigate disruptions, healthcare systems including VA rapidly pivoted to virtual care to maintain continuous services.^15^ Yet, this abrupt pivot caused concern that some disadvantaged groups would be left behind.

Racial-ethnic minority groups have historically borne the brunt of the “digital divide,” a phenomenon that refers to disparities in access to health information technologies and virtual care.^16^ While some studies indicate a digital divide in VA,^17^ other studies indicate that veterans of color access digital health technologies more than White veterans.^18, 19^ Little is known about whether disparities in use of virtual specialty pain care were exacerbated for veterans of color during the start of COVID-19.

There is a substantial literature documenting suboptimal pain care for minoritized racial and ethnic patients.^20–22^ Black veterans are less likely than White veterans to receive pain clinic visits,^23^ and compared to their counterparts, Black and Hispanic/Latinx veterans are more likely to use emergency and urgent care to address pain. In studies outside of VA, patients of color are less likely to receive opioids or analgesics than White patients,^24–26^ have shorter visit times with pain care providers,^26^ and generally receive less aggressive pain treatment than White patients despite similarities in pain severity.^27^ Medical trainees and laypeople falsely believe that biological pain differences exist between Black and White patients and Black patients are less likely to be recommended optimal pain care.^28, 29^ Given both historical and ongoing injustices in pain care and the potential for virtual care disparities, it is of the utmost importance to identify whether disparities in virtual pain care exist in the post-COVID-19 world.

The purpose of this study is to determine whether disparities in receipt of specialty pain care were exacerbated for racial-ethnic minority groups during the onset of COVID-19, with a focus on virtual care. Our study builds on research examining racial-ethnic disparities in telehealth use during the pandemic and is the first in the field to examine disparities in utilization of pain care specialty services during COVID-19 at a national level.

## METHODS

### Design and Participants

This study was designated as quality improvement by the institutional review board of record (see Supplementary Appendix A). This was a retrospective cohort study using data from the VA Corporate Data Warehouse. We first identified all patients who had ≥ 1 primary care encounter as indicated by specific VA stop codes, or 3-digit identifiers unique to VA that are used to identify types of outpatient encounters and inpatient professional services provided by specific groups (e.g., primary care, mental health). Patients entered one of two cohorts if they were active during either (1) the year prior to COVID-19 (3/01/19–2/29/20; index date 1) or (2) one year earlier (3/01/18–2/28/19; index date 2). Index date 1 was established to examine specialty pain care use during the initial onset of COVID-19, and index date 2 was established as a comparison cohort to assess specialty pain care use during a parallel, 6-month window.

Only patients with documented chronic pain in the year prior to their index date were included the final analytic cohort. In the current study, we adapted chronic pain and pain diagnostic categorizations  from Tian et al. and Mayhew et al.^30, 31^ Patients met chronic pain criteria if they had ≥ 2 documented diagnoses 90–365 days apart within one of the following diagnostic clusters: (1) back pain; (2) neck pain; (3) limb/extremity pain, joint pain and non-systemic, non-inflammatory arthritic disorders; (4) fibromyalgia; (5) headache; (6) orofacial, ear, and temporomandibular disorder pain; (7) abdominal and bowel pain; (8) urogenital, pelvic, and menstrual pain; (9) musculoskeletal chest pain; (10) neuropathy; (11) systemic disorders or diseases causing pain; or (12) other painful conditions).

### Main Measures

#### Outcome Measure: Utilization of Specialty Pain Care (Telehealth/In-person/Any)

The primary outcome was utilization of VA specialty pain care recorded between 5/01/19–11/01/19 (pre-COVID period) and 5/01/20–11/01/20 (COVID-era period). This outcome was binary (i.e., any encounter vs. no encounters) and period-specific.

We defined “telehealth” as “the use of electronic information and telecommunications technologies to support and promote long-distance clinical health care.”^33^ As such, our definition of telehealth includes both telephone and video visits; specifically, we include any visit with a primary VA stop code of 420 (i.e., indicating specialty pain care receipt) and a secondary VA stop code of 179, 690, 692, 693, 723, or 724 as a video visit (see Supplementary Appendix B for definitions). Telephone encounters are any visit with a primary or secondary VA stop code of 420 and include the string “telephone.”

We also examined the outcomes of in-person and any specialty pain care. “In-person care” is defined as any visit that does not meet the definition of telehealth. We excluded the following VA stop codes from our “in-person” pain care variable: 683, 684, 685, and 719 (see Supplementary Appendix B for definitions). Following existing guidance,^32^ we defined “any specialty pain care” as any visit with a primary or secondary VA stop code of 420 (see Limitations, p. 14 for limitations in using the 420 stop code).

#### Independent Measures: Racial and Ethnic Identity

Our independent measures were racial and ethnic identity. We followed best practices for classifying racial-ethnic identity to both maximize data accuracy and minimize researchers’ assumptions about patients’ race and ethnicity.^34^ Using VA administrative data for “patient race,” we first created separate binary variables to reflect White, Black or African American, American Indian/Alaska Native (AI/AN), Native Hawaiian or Other Pacific Islander (NHOPI), and Asian race. Then, we combined these separate race variables into a single variable with the mutually exclusive categories of White, Black, AI/AN, NHOPI, Asian, multiple races reported, or unknown. Individuals were assigned to a race category if they had at least one indicator for any individual category in the original “patient race” variable. Individuals with indicators in multiple race categories were designated as “multiple races reported.” Any individual who had a value of “0” across all race values were designated as “unknown.”

We used a separate VA administrative data variable for “patient ethnicity” to define our ethnicity variable. We designated individuals as “Hispanic or Latinx” if they had an indicator for “Hispanic or Latino” and all others as “Non-Hispanic or Latinx” if there was no indicator for Hispanic or Latino ethnicity present.

#### Covariates

As our goal was to identify disparities, we chose covariates based on evidence of existing relationships with the exposure variable (patient race or ethnicity) and our outcome of interest (i.e., utilization of specialty pain care, and specifically telehealth) in extant literature: age in years, gender (man/woman),^35, 36^ and rurality (urban/rural/highly rural/insular island).^32, 37^ All covariates were measured at index date for each cohort.

### Analytic Approach

We first examined descriptive differences between both cohorts. We next used a quasi-Poisson regression modeling approach to estimate the probability of receiving any specialty pain care during each period, adjusted for days of follow-up. For each of our three outcomes (i.e., specialty pain care via telehealth, in-person specialty pain care, or any specialty pain care), we fit separate models for race and ethnicity. For both race and ethnicity models, we fit two adjusted models including (1) rurality, and (2) rurality, age, and gender.

## KEY RESULTS

### Characteristics of Study Cohorts and Type of Care

There were 1,649,053 COVID-era patients with chronic pain who met inclusion criteria. Individuals included in the pre-COVID cohort were also included in the COVID-era cohort if they met inclusion criteria. Our cohorts were similar across all demographic variables, with differences not exceeding more than one percentage point in most cases (see Table 1). All patients were included in subsequent regressions.

**Table 1:** Characteristics of all VA Patients with Chronic Pain Stratified by Cohort Period (Includes all Patients Diagnosed with Chronic Pain Across Period Cohorts)

**Population characteristics stratified by cohort period**	**Pre-COVID cohort**^**†**^** (*n* = 1,536,934)**	**COVID-era cohort**^‡^ **(*n* = 1,649,053)**
	***N* (%)**	***N* (%)**
**Age, mean (SD)**	60.6 (14.6)	60.7 (14.7)
**Age (years)**		
< 30	42,290 (2.8)	44,416 (2.7)
30–39	125,920 (8.2)	140,514 (8.5)
40–49	167,554 (10.9)	181,963 (11.0)
50–59	291,013 (18.9)	304,993 (18.5)
60–69	488,612 (31.8)	473,976 (28.7)
70–79	300,679 (19.6)	375,793 (22.8)
80–89	101,077 (6.6)	106,885 (6.5)
90 +	19,780 (1.3)	20,500 (1.2)
**Race**	(*n* = 92,039 missing)	(*n* = 101,092 missing)
White	1,070,947 (69.7)	1,140,789 (69.2)
Black	316,527 (20.6)	343,451 (20.8)
Asian	15,253 (1.0)	17,664 (1.1)
Native Hawaiian or Other Pacific Islander	14,366 (0.9)	15,753 (1.0)
American Indian or Alaska Native	12,808 (0.8)	13,932 (0.8)
2 + races	14,994 (1.0)	16,372 (1.0)
**Ethnicity**	(*n* = 94,772 missing)	(*n* = 105,390)
Hispanic/Latinx	105,358 (6.9)	117,251 (7.1)
Non-Hispanic/Latinx	1,336,804 (87.0)	1,426,412 (86.5)
**Gender**—women	166,955 (10.9)	186,233 (11.3)
**Rural/urban dwelling**		
Urban	979,137 (63.7)	1,063,021 (64.5)
Rural	477,704 (31.1)	512,247 (31.1)
Highly rural	59,954 (3.9)	62,828 (3.8)
Insular island	1241 (0.1)	1464 (0.1)
**Marital status**		
Divorced/separated	428,222 (27.9)	451,201 (27.4)
Married	842,704 (54.8)	911,516 (55.3)
Never married/single	177,953 (11.6)	195,289 (11.9)
Widowed	73,603 (4.8)	73,424 (4.5)
Unknown	14,452 (0.9)	17,623 (1.1)
**Pain diagnostic category (in order of prevalence in sample)**		
1. Limb/extremity pain, joint pain, etc	854,767 (55.6)	928,420 (56.3)
2. Back pain	590,802 (38.4)	647,390 (39.3)
3. Neuropathy	211,895 (13.8)	227,053 (13.8)
4. Other painful conditions^§^	192,267 (12.5)	214,387 (13.0)
5. Neck pain	158,820 (10.3)	186,128 (11.3)
6. Abdominal and bowel pain	108,856 (7.1)	120,132 (7.3)
7. Headache	70,142 (4.6)	83,548 (5.1)
8. Musculoskeletal chest pain	55,244 (3.6)	58,562 (3.6)
9. Fractures, contusions, sprains, and strains	46,958 (3.1)	51,638 (3.1)
10. Systemic disorders or diseases causing pain	38,808 (2.5)	41,813 (2.5)
11. Fibromyalgia	29,863 (1.9)	27,395 (1.7)
12. Urogenital, pelvic, and menstrual pain	14,061 (0.9)	15,701 (1.0)
13. Orofacial, ear, and temporomandibular disorder pain	4316 (0.3)	5003 (0.3)

^†^Pre-COVID patients were initially seen in primary care for chronic pain from 3/1/18 to 2/28/19 and followed up from 5/1/19 to 11/1/19

^‡^COVID-era patients were initially seen in primary care for chronic pain from 3/1/19 to 2/29/20 and followed up from 5/1/20 to 11/1/20

^§^E.g., sickle cell disease, complex regional pain syndrome, systemic lupus erythematosus, acquired deformities, spinal cord injury, Lyme disease

**Figure 1:**
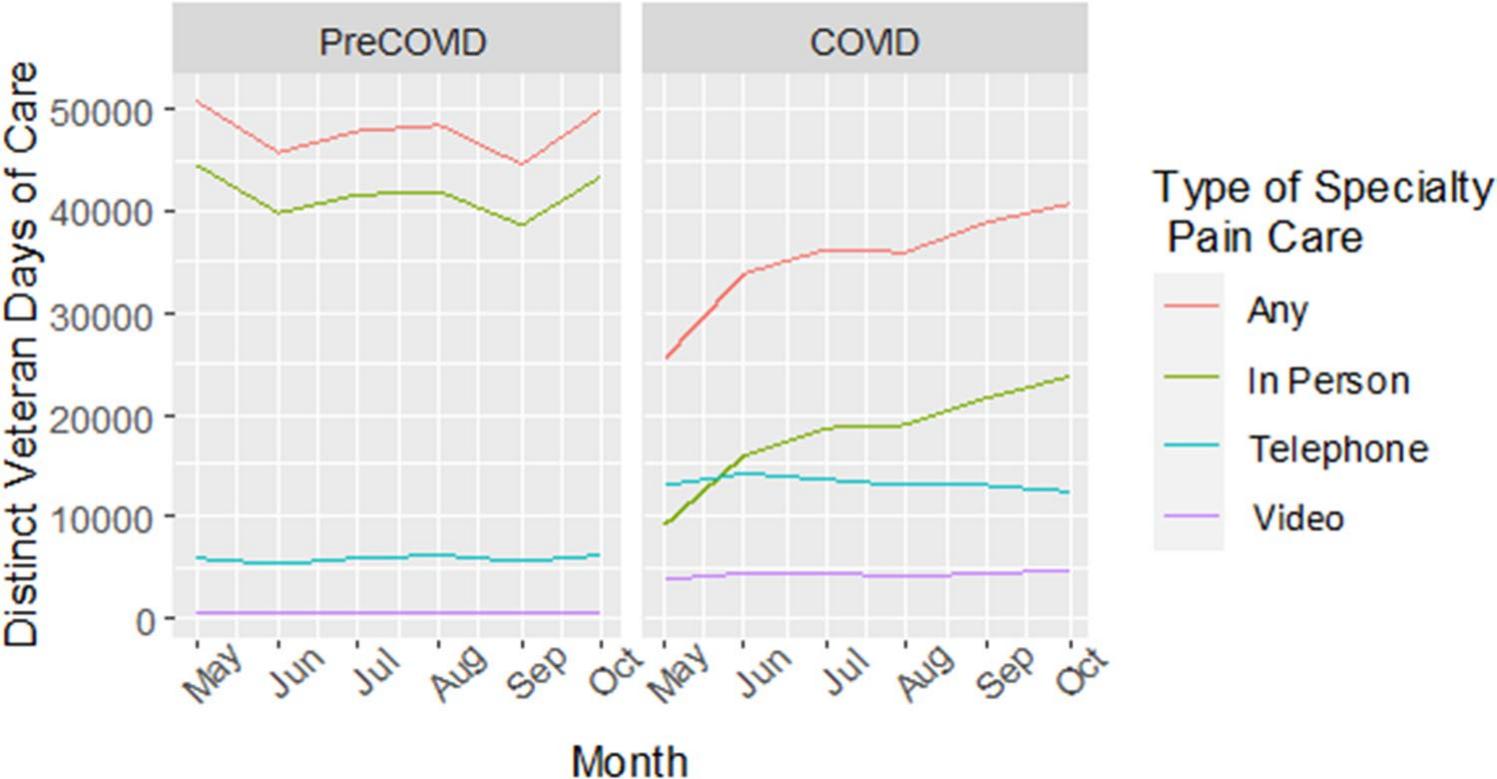
Line graph indicating number of distinct Veteran-days of care per month during the pre-COVID and COVID eras by type of specialty pain care visit (i.e., any pain specialty care, in-person pain specialty care, pain specialty care via telehealth).

Table 2 presents characteristics of individuals across cohorts who had any specialty pain care encounter. Across both cohorts, individuals who had any specialty pain care encounter were mostly White and non-Hispanic/Latinx men aged 60–69. Most individuals across both cohorts resided in an urban setting and were married or cohabiting. The top three pain diagnoses across cohorts were back pain, limb/extremity or joint pain, and other painful conditions. Both prior to and during COVID-19, most specialty pain care encounters were in-person, followed by telephone encounters, then video encounters (see Fig. 1).

**Table 2:** Characteristics of VA Patients with Chronic Pain who had a Specialty Pain Care Encounter Stratified by Receipt of in-person, Telephone, or Video Specialty Pain Care Encounter (Column Percentages Represented)

Population characteristics stratified by type of specialty pain care encounter	Pre-COVID cohort (*n* = 100,065)				COVID-era cohort (*n* = 82,584)			
	**Any encounter (*n* = 100,065)**	**In-person (*n* = 96,533)**	**Telephone (*n* = 18,262)**	**Video (*n* = 1,897)**	**Any encounter (*n* = 82,584)**	**In-person (*n* = 59,832)**	**Telephone (*n* = 38,837)**	**Video (*n* = 12,051)**
	***N* (Col %)**	***N* (Col %)**	***N* (Col %)**	***N* (Col %)**	***N* (Col %)**	***N* (Col %)**	***N* (Col %)**	***N* (Col %)**
**Age, mean (SD)**	58.18 (13.01)	58.11 (13.02)	59.30 (12.51)	58.30 (12.96)	58.49 (13.13)	58.58 (13.13)	59.10 (12.90)	55.39 (13.22)
**Age (years)**								
< 30	2002 (2.0)	1953 (2.0)	241 (1.3)	35 (1.8)	1556 (1.9)	1085 (1.8)	650 (1.7)	325 (2.7)
30–39	8567 (8.6)	8319 (8.6)	1330 (7.3)	174 (9.2)	7172 (8.7)	5128 (8.6)	3094 (8.0)	1451 (12.0)
40–49	13,946 (13.9)	13,525 (14.0)	2284 (12.5)	243 (12.8)	11,377 (13.8)	8318 (13.9)	4991 (12.9)	2036 (16.9)
50–59	24,623 (24.6)	23,791 (24.6)	4430 (24.3)	461 (24.3)	19,740 (23.9)	14,248 (23.8)	9154 (23.6)	3210 (26.6)
60–69	32,131 (32.1)	30,914 (32.0)	6269 (34.3)	623 (32.8)	25,004 (30.3)	18,057 (30.2)	12,269 (31.6)	3223 (26.7)
70–79	15,486 (15.5)	14,876 (15.4)	3055 (16.7)	297 (15.7)	15,010 (18.2)	10,966 (18.3)	7324 (18.9)	1596 (13.2)
80–89	3012 (3.0)	2866 (3.0)	607 (3.3)	60 (3.2)	2508 (3.0)	1867 (3.1)	1254 (3.2)	191 (1.6)
90 +	297 (0.3)	288 (0.3)	46 (0.3)	4 (0.2)	216 (0.3)	162 (0.3)	101 (0.3)	19 (0.2)
**Race**								
White	70,516 (70.5)	67,796 (70.2)	13,596 (74.4)	1532 (80.8)	58,253 (70.5)	42,371 (70.8)	27,810 (71.6)	8203 (68.1)
Black	19,893 (19.9)	19,408 (20.1)	3017 (16.5)	210 (11.1)	16,064 (19.5)	11,550 (19.3)	7169 (18.5)	2531 (21.0)
Asian	990 (1.0)	975 (1.0)	130 (0.7)	4 (0.2)	836 (1.0)	621 (1.0)	326 (0.8)	167 (1.4)
NHOPI^†^	930 (1.0)	905 (0.9)	129 (0.7)	14 (0.7)	809 (1.0)	543 (0.9)	404 (1.0)	134 (1.1)
AI/AN^‡^	867 (0.9)	834 (0.9)	163 (0.9)	20 (1.1)	682 (0.8)	508 (0.8)	310 (0.8)	96 (0.8)
2 + races	1048 (1.0)	1012 (1.0)	174 (1.0)	19 (1.0)	859 (1.0)	605 (1.0)	421 (1.1)	126 (1.0)
Unknown/missing	5821 (5.8)	5603 (5.8)	1053 (5.8)	98 (5.2)	5081 (6.2)	3634 (6.1)	2397 (6.2)	794 (6.6)
**Ethnicity**								
Hispanic/Latinx	7495 (7.5)	7338 (7.6)	910 (5.0)	79 (4.2)	5912 (7.2)	3967 (6.6)	3006 (7.7)	1067 (8.9)
Non-Hispanic/Latinx	85,942 (85.9)	82,812 (85.8)	16,078 (88.0)	1721 (90.7)	70,860 (85.8)	51,706 (86.4)	33,116 (85.3)	10,117 (84.0)
Unknown/missing	6628 (6.6)	6383 (6.6)	1274 (7.0)	97 (5.1)	5812 (7.0)	4159 (7.0)	2715 (7.0)	867 (7.2)
**Gender**								
Women	13,964 (14.0)	13,553 (14.0)	2214 (12.1)	244 (12.9)	11,822 (14.3)	8517 (14.2)	5197 (13.4)	2266 (18.8)
**Rural/urban dwelling**								
Urban	68,578 (68.5)	66,400 (68.8)	12,123 (66.4)	990 (52.2)	56,038 (67.9)	40,129 (67.1)	26,590 (68.5)	8867 (73.6)
Rural	27,975 (28.0)	26,827 (27.8)	5484 (30.0)	763 (40.2)	23,651 (28.6)	17,558 (29.3)	10,836 (27.9)	2875 (23.9)
Highly rural	3455 (3.2)	2966 (3.1)	584 (3.2)	134 (7.1)	2540 (3.1)	1925 (3.2)	1202 (3.1)	240 (2.0)
Insular island	15 (0.0)	13 (0.0)	3 (0.0)	0 (0.0)	50 (0.1)	21 (0.0)	43 (0.1)	24 (0.2)
**Marital status**								
Divorced/separated	30,114 (30.1)	36,614 (38.0)	5624 (30.8)	547 (28.8)	24,668 (29.8)	22,357 (37.3)	11,816 (30.4)	3555 (29.5)
Married or cohabiting	53,969 (53.9)	52,054 (53.9)	9835 (53.9)	1111 (58.6)	44,818 (54.3)	32,719 (54.7)	20,879 (53.8)	6587 (54.7)
Never married/single	11,688 (11.7)	11,323 (11.8)	1989 (10.9)	164 (8.6)	9640 (11.7)	6947 (11.6)	4487 (11.5)	1505 (12.5)
Widowed	3500 (3.5)	3356 (3.5)	689 (3.8)	65 (3.4)	2758 (3.3)	1986 (3.3)	1334 (3.4)	317 (2.6)
Unknown	794 (0.8)	774 (0.8)	125 (0.7)	10 (0.5)	700 (0.8)	497 (0.8)	321 (0.8)	45 (0.4)
**Pain diagnostic category (in order of prevalence in sample)**								
Back pain	64,397 (64.4)	62,177 (64.4)	12,739 (69.8)	1083 (57.1)	54,941 (66.5)	39,905 (66.7)	26,771 (68.9)	8054 (66.8)
Limb/extremity pain, joint pain, etc.^§^	55,234 (55.2)	53,436 (55.4)	9859 (54.0)	981 (51.7)	46,639 (56.5)	34,114 (57.0)	21,896 (56.4)	6908 (57.3)
Other painful conditions^§^	23,344 (23.2)	22,429 (23.2)	4670 (25.6)	575 (30.3)	20,833 (25.2)	14,738 (24.6)	10,506 (27.1)	3551 (29.5)
Neck pain	20,450 (20.4)	19,805 (20.5)	3837 (21.0)	361 (19.0)	18,369 (22.2)	13,467 (22.5)	8872 (22.8)	2734 (22.7)
Neuropathy	12,072 (12.1)	11,551 (12.0)	2353 (12.9)	286 (15.1)	10,318 (12.5)	7453 (12.5)	5077 (13.1)	1423 (11.8)
Headache	7585 (7.6)	7406 (7.7)	1148 (6.3)	111 (5.9)	6869 (8.3)	5165 (8.6)	2944 (7.6)	1227 (10.2)
Abdominal and bowel pain	6989 (7.0)	6755 (7.0)	1274 (7.0)	131 (6.9)	6287 (7.6)	4593 (7.7)	2946 (7.6)	924 (7.7)
Fibromyalgia	5113 (5.1)	4991 (5.2)	847 (4.6)	88 (4.6)	3536 (4.3)	2583 (4.3)	1689 (4.3)	674 (5.6)
Musculoskeletal chest pain	3848 (3.8)	3724 (3.9)	671 (3.7)	62 (3.3)	3301 (4.0)	2451 (4.1)	1541 (4.0)	463 (3.8)
Fractures, contusions, sprains, and strains	3467 (3.5)	3366 (3.5)	635 (3.5)	62 (3.3)	3052 (3.7)	2239 (3.7)	1434 (3.7)	445 (3.7)
Systemic disorders or diseases causing pain	2444 (2.4)	2342 (2.4)	462 (2.5)	77 (4.1)	2229 (2.7)	1586 (2.7)	1072 (2.8)	357 (3.0)
Urogenital, pelvic, and menstrual pain	1068 (1.1)	1035 (1.1)	184 (1.0)	17 (0.9)	999 (1.2)	722 (1.2)	470 (1.2)	171 (1.4)
Orofacial, ear, and temporomandibular disorder pain	554 (0.6)	537 (0.6)	107 (0.6)	8 (0.4)	514 (0.6)	400 (0.7)	237 (0.6)	77 (0.6)

Types of Encounters were not Mutually Exclusive, i.e., an Individual in the Study Cohort Could Have Received Both in-person and Telephone Care in the 6-month Follow-up Period

^†^*NHOPI* Native Hawaiian or Other Pacific Islander

^‡^*AI/AN* American Indian or Alaska Native

^§^Limb/extremity pain, joint pain, and non-systemic, non-inflammatory arthritic disorders

^¶^Other painful conditions include sickle cell disease, complex regional pain syndrome, systemic lupus erythematosus, acquired deformities, spinal cord injury, Lyme disease

### Pain Care Via Telehealth

**Figure 2:**
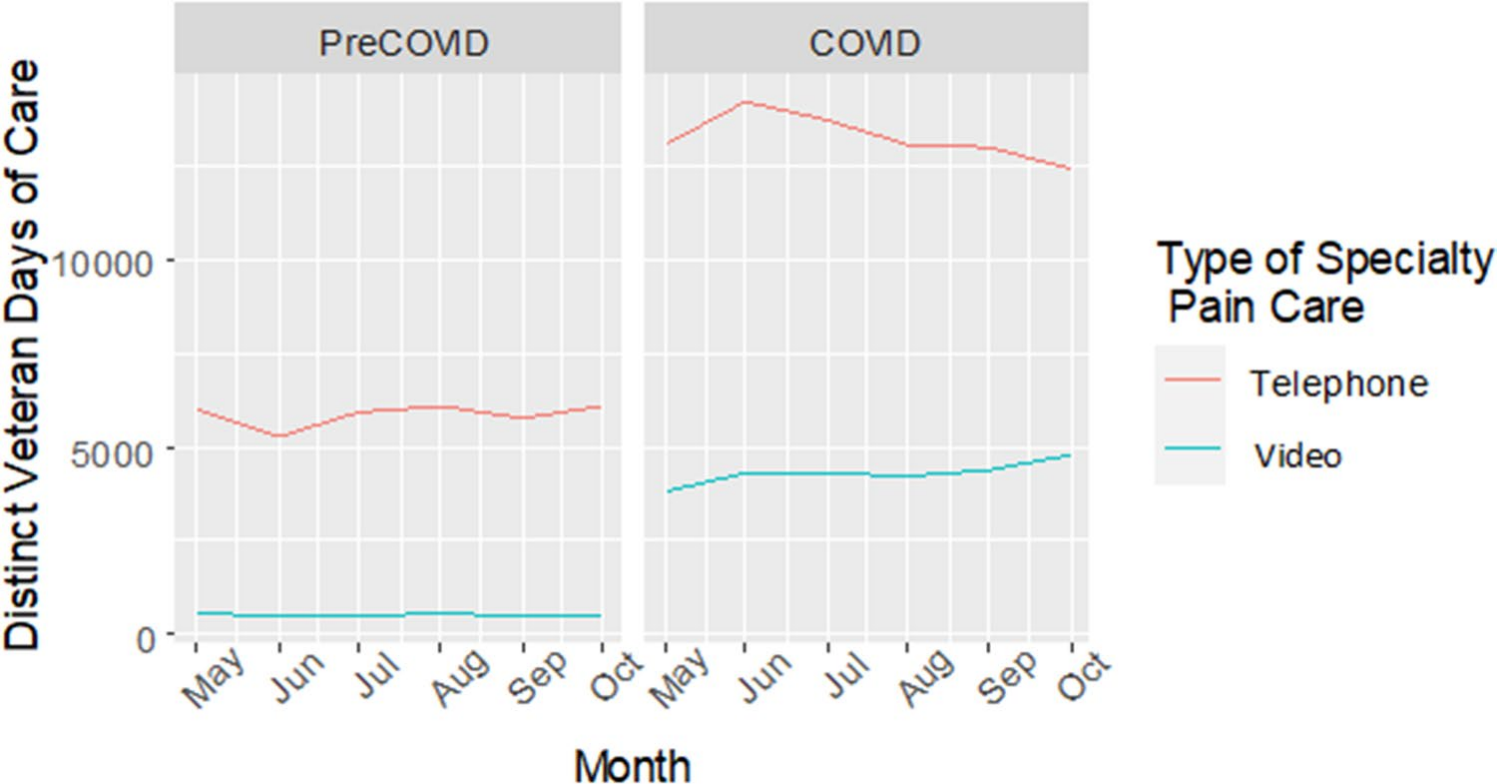
Line graph indicating number of distinct Veteran-days of care per month during the pre-COVID and COVID eras by type of telehealth specialty pain care visit (i.e., telephone vs. video encounters).

**Figure 3:**
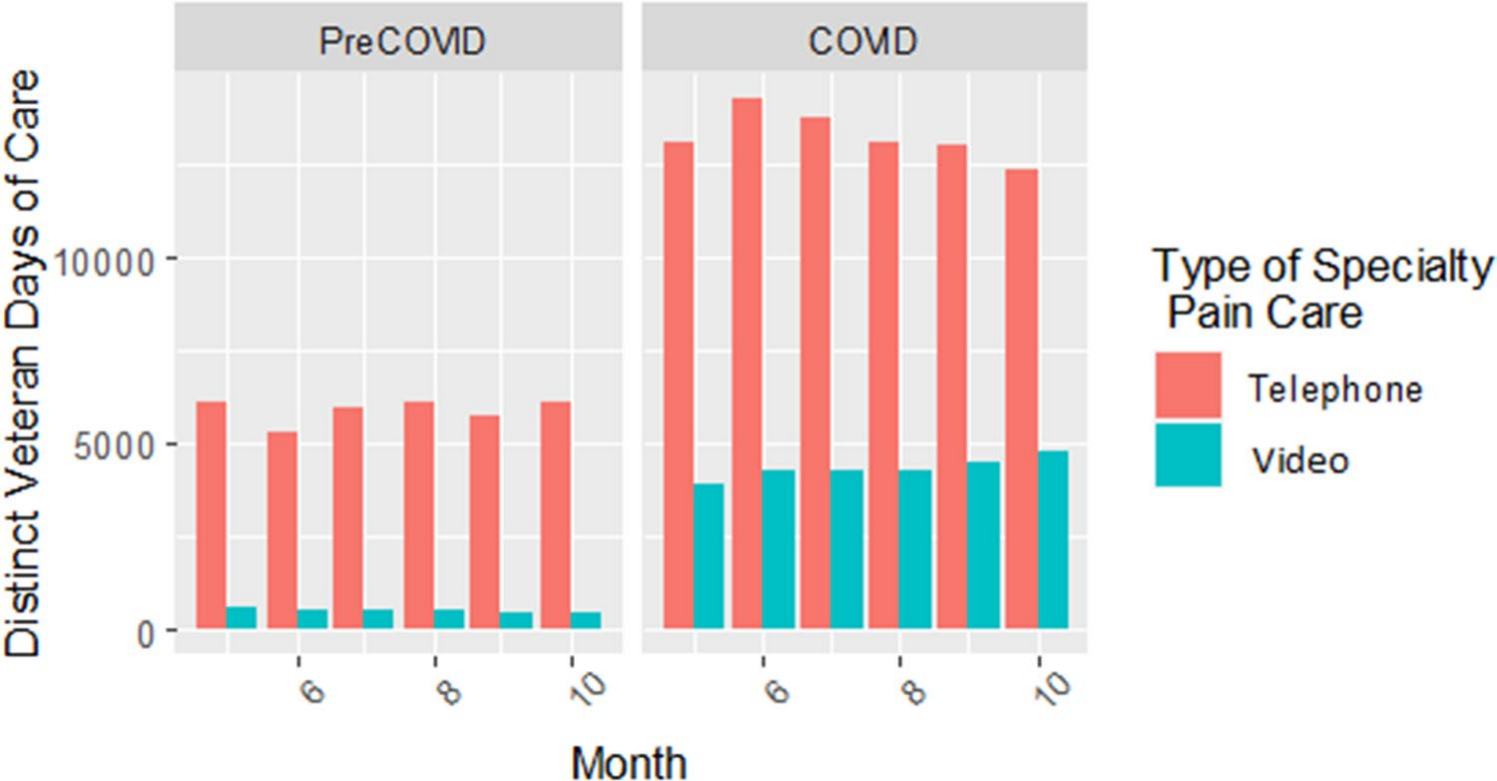
Bar chart indicating number of distinct Veteran-days of care per month during the pre-COVID and COVID eras by type of telehealth specialty pain care visit (i.e., telephone vs. video encounters).

Results from quasi-Poisson models for telehealth specialty pain encounters are given in Table 3 and are visualized in Figs. 2 and 3. Prior to COVID-19, Black veterans were 36% less likely than White veterans to have a telehealth specialty pain encounter (pre-COVID adjusted RR = 0.64, 95% CI [0.62, 0.67]); Asian veterans were 37% less likely than White veterans to have a telehealth specialty pain encounter (pre-COVID adjusted RR = 0.63, 95% CI [0.54, 0.75]); and veterans identifying as NHOPI were 29% less likely than White veterans to have a telehealth specialty pain encounter (pre-COVID adjusted RR = 0.71, 95% CI [0.60, 0.83]). Veterans identifying as AI/AN were equally likely as White veterans to use specialty pain care via telehealth (pre-COVID adjusted RR = 0.98, 95% CI [0.85, 1.13]).

**Table 3:** Risk Ratios and 95% Confidence Intervals from Quasi-Poisson Models for Receipt of Pain Care via Telehealth (Telephone and Video Encounters) by Race and Ethnicity Separately

Predictors	Pre-COVID			COVID-era		
	**Unadjusted model 1**	**Model 2: rurality included**	**Model 3: rurality, age, gender included**	**Unadjusted model 1**	**Model 2: rurality included**	**Model 3: rurality, age, gender included**
	**Estimate (95% CI)**	**Estimate (95% CI)**	**Estimate (95% CI)**	**Estimate (95% CI)**	**Estimate (95% CI)**	**Estimate (95% CI)**
**Race**						
White	Ref	Ref	Ref	Ref	Ref	Ref
Black	0.73 (0.70, 0.76)	0.71 (0.69, 0.74)	0.64 (0.62, 0.67)	0.89 (0.87, 0.91)	0.84 (0.82, 0.86)	0.75 (0.73, 0.77)
Asian	0.64 (0.54, 0.76)	0.66 (0.55, 0.78)	0.63 (0.54, 0.75)	0.87 (0.80, 0.96)	0.87 (0.79, 0.95)	0.81 (0.74, 0.89)
NHOPI*	0.71 (0.60, 0.84)	0.74 (0.62, 0.87)	0.71 (0.60, 0.83)	1.07 (0.98, 1.17)	1.03 (0.94, 1.12)	0.97 (0.88, 1.06)
AI/AN^†^	1.03 (0.89, 1.19)	1.03 (0.89, 1.20)	0.98 (0.85, 1.13)	0.91 (0.83, 1.01)	0.93 (0.84, 1.03)	0.87 (0.79, 0.96)
2 + races	0.93 (0.81, 1.08)	0.93 (0.80, 1.07)	0.89 (0.78, 1.03)	1.03 (0.95, 1.13)	1.00 (0.92, 1.10)	0.94 (0.86, 1.03)
**Ethnicity**						
Non-Hispanic/Latinx	Ref	Ref	Ref	Ref	Ref	Ref
Hispanic/Latinx	0.71 (0.67, 0.76)	0.70 (0.66, 0.76)	0.70 (0.66, 0.75)	1.12 (1.08, 1.16)	1.07 (1.03, 1.11)	1.06 (1.02, 1.09)

^*^NHOPI Native Hawaiian or Other Pacific Islander

^†^*AI/AN* American Indian or Alaska Native

Disparities improved during the start of the pandemic but did not disappear. In our adjusted model, Black veterans were 25% less likely than White veterans to use telehealth during the start of COVID-19 (RR = 0.75, 95% CI [0.73, 0.77]) and Asian veterans were 19% less likely to use telehealth (RR = 0.81, 95% CI [0.74, 0.89]). Whereas no AI/AN-White disparity in telehealth use existed prior to COVID-19, veterans identifying as AI/AN were 13% less likely to use telehealth compared to White veterans at the start of COVID-19 (RR = 0.87, 95% CI [0.79, 0.96]). In contrast, while veterans identifying as NHOPI were less likely to use telehealth compared to White veterans prior to COVID, this disparity disappeared when COVID-19 began (RR = 0.97, 95% CI [0.88, 1.06]).

Similarly, Hispanic/Latinx veterans were 30% less likely than non-Hispanic/Latinx veterans to use telehealth for pain care prior to COVID-19 (adjusted RR = 0.70, 95% CI [0.66, 0.75]). At the start of COVID-19, this trend reversed, and Hispanic/Latinx veterans were 6% more likely than non-Hispanic/Latinx veterans to use telehealth in our adjusted model (RR = 1.06, 95% CI [1.02, 1.09]).

### In-person Pain Care

Results from models for in-person pain care are given in Table 4. We found effects for both race and ethnicity in use of in-person care both prior and during the first months of COVID-19. Black veterans were 18% less likely than White veterans to receive in-person care prior to COVID-19, with disparities confounded by rurality, age, and gender (pre-COVID adjusted RR = 0.82, 95% CI [0.81, 0.83]). When COVID-19 began, Black veterans were 22% less likely than White Veterans to receive in-person care (COVID-era adjusted RR = 0.78, 95% CI [0.77, 0.80]). Similarly, Asian veterans were 7% less likely than White veterans to receive in-person pain care prior to COVID-19 in adjusted models (pre-COVID adjusted RR = 0.93, 95% CI [0.88, 0.99]) and 9% less likely at the beginning of COVID-19 (COVID-era adjusted RR = 0.91, 95% CI [0.84, 0.99]). While individuals who identify as NHOPI were equally likely as White veterans to receive in-person pain care prior to COVID-19 (pre-COVID adjusted RR = 0.96, 95% CI [0.90, 1.02]), they were 11% less likely to receive in-person care during the beginning of COVID-19 (COVID-era adjusted RR = 0.89, 95% CI [0.82, 0.97]).

**Table 4:** Risk Ratios and 95% Confidence Intervals from Quasi-Poisson Models for Receipt of In-person Pain Care by Race and Ethnicity Separately

Predictors	Pre-COVID			COVID-era		
	**Unadjusted model 1**	**Model 2: rurality included**	**Model 3: rurality, age, gender included**	**Unadjusted model 1**	**Model 2: rurality included**	**Model 3: rurality, age, gender included**
	**Estimate (95% CI)**	**Estimate (95% CI)**	**Estimate (95% CI)**	**Estimate (95% CI)**	**Estimate (95% CI)**	**Estimate (95% CI)**
**Race**						
White	Ref	Ref	Ref	Ref	Ref	Ref
Black	0.97 (0.95, 1.07)	0.92 (0.91, 0.93)	0.82 (0.81, 0.83)	0.91 (0.89, 0.92)	0.88 (0.86, 0.90)	0.78 (0.77, 0.80)
Asian	1.01 (0.95, 1.07)	1.00 (0.94, 1.07)	0.93 (0.88, 0.99)	0.95 (0.88, 1.02)	0.98 (0.90, 1.06)	0.91 (0.84, 0.99)
NHOPI*	1.00 (0.93, 1.06)	1.01 (0.95, 1.08)	0.96 (0.90, 1.02)	0.93 (0.85, 1.01)	0.95 (0.87, 1.03)	0.89 (0.82, 0.97)
AI/AN^†^	1.03 (0.96, 1.10)	1.04 (0.98, 1.12)	0.97 (0.91, 1.04)	0.98 (0.90, 1.07)	1.00 (0.90, 1.06)	0.93 (0.86, 1.02)
2 + races	1.07 (1.00, 1.13)	1.04 (0.98, 1.11)	0.98 (0.92, 1.04)	0.99 (0.92, 1.08)	0.98 (0.90, 1.06)	0.92 (0.85, 1.00)
**Ethnicity**						
Non-Hispanic/Latinx	Ref	Ref	Ref	Ref	Ref	Ref
Hispanic/Latinx	1.12 (1.10, 1.15)	1.08 (1.05, 1.10)	1.06 (1.04, 1.09)	0.93 (0.90, 0.96)	0.91 (0.88, 0.94)	0.90 (0.87, 0.93)

^*^NHOPI Native Hawaiian or Other Pacific Islander

^†^*AI/AN* American Indian or Alaska Native

Hispanic/Latinx veterans were more likely to receive in-person pain care relative to non-Hispanic/Latinx veterans prior to COVID-19 (pre-COVID adjusted RR = 1.06, 95% CI [1.04, 1.09]). Yet, at the start of COVID-19, Hispanic/Latinx veterans were 10% less likely than non-Hispanic/Latinx veterans to receive in-person care (COVID-era adjusted RR = 0.90, 95% CI [0.87, 0.93]).

### Any Specialty Pain Care

There was an overall raw decrease of 17,481 specialty care encounters from the pre-COVID to COVID-era cohort. We found an effect for race on any specialty pain care for Black and Asian veterans (see Table 5). Prior to COVID-19, there was a small observed disparity in receipt of any specialty pain care between Black and White veterans, with Black veterans being 5% less likely to receive any specialty pain care compared to White veterans (unadjusted RR = 0.95, 95% CI [0.94, 0.97]). Once we adjusted for rurality, age, and gender, Black veterans were 19% less likely than White veterans to receive any specialty pain care pre-COVID-19 (adjusted RR = 0.81, 95% CI [0.80, 0.83]). The Black-White disparity in receipt of any specialty pain care was larger in COVID-era adjusted models (unadjusted RR = 0.92, 95% CI [0.90, 0.93]; adjusted RR = 0.79, 95% CI [0.77, 0.80]). Asian veterans were 9% less likely to receive any specialty pain care compared to White veterans in adjusted models during the pre-COVID era (adjusted RR = 0.91, 95% CI [0.86, 0.97]). This disparity was larger during COVID-19 onset, at 12% less likelihood (adjusted RR = 0.88, 95% CI [0.82, 0.94)].

**Table 5:** Risk Ratios and 95% Confidence Intervals From Quasi-Poisson Models for Receipt of any Specialty Pain Care by Race and Ethnicity Separately

Predictors	Pre-COVID			COVID-era		
	**Unadjusted model 1**	**Model 2: rurality included**	**Model 3: rurality, age, gender included**	**Unadjusted model 1**	**Model 2: rurality included**	**Model 3: rurality, age, gender included**
	**Estimate (95% CI)**	**Estimate (95% CI)**	**Estimate (95% CI)**	**Estimate (95% CI)**	**Estimate (95% CI)**	**Estimate (95% CI)**
**Race**						
White	Ref	Ref	Ref	Ref	Ref	Ref
Black	0.95 (0.94, 0.97)	0.91 (0.89, 0.92)	0.81 (0.80, 0.83)	0.92 (0.90, 0.93)	0.88 (0.86, 0.90)	0.79 (0.77, 0.80)
Asian	0.99 (0.93, 1.05)	0.98 (0.92, 1.04)	0.91 (0.86, 0.97)	0.93 (0.87, 0.99)	0.95 (0.89, 1.01)	0.88 (0.82, 0.94)
NHOPI*	0.98 (0.92, 1.05)	1.00 (0.94, 1.07)	0.95 (0.89, 1.01)	1.01 (0.94, 1.08)	1.00 (0.93, 1.07)	0.94 (0.88, 1.01)
AI/AN^†^	1.06 (0.96, 1.10)	1.04 (0.98, 1.11)	0.97 (0.91, 1.04)	0.96 (0.89, 1.03)	0.97 (0.90, 1.05)	0.91 (0.85, 0.98)
2 + races	1.06 (1.00, 1.13)	1.04 (0.98, 1.10)	0.98 (0.92, 1.04)	1.03 (0.96, 1.10)	1.01 (0.94, 1.07)	0.95 (0.88, 1.01)
**Ethnicity**						
Non-Hispanic/Latinx	Ref	Ref	Ref	Ref	Ref	Ref
Hispanic/Latinx	1.11 (1.08, 1.13)	1.06 (1.04, 1.09)	1.05 (1.02, 1.07)	1.01 (0.99, 1.04)	0.98 (0.96, 1.01)	0.97 (0.95, 1.00)

^*^NHOPI Native Hawaiian or Other Pacific Islander

^†^*AI/AN* American Indian or Alaska Native

Individuals of Hispanic/Latinx ethnicity utilized any specialty pain care at higher rates than non-Hispanic/Latinx individuals before COVID-19 (pre-COVID unadjusted RR = 1.11, 95% CI [1.08, 1.13]), but this difference was attenuated when adjusting for age, gender, and rurality (pre-COVID adjusted RR = 1.05, 95% CI [1.02, 1.07]). Following COVID-19 onset, Hispanic/Latinx veterans were equally likely to receive any specialty pain care as non-Hispanic/Latinx veterans (COVID-era adjusted RR = 0.97, 95% CI [0.95, 1.00]).

## DISCUSSION

Disparities in use of telehealth specialty pain care lessened during the onset of the pandemic in the context of increases in telehealth use generally, but disparities persisted for certain racial groups. Ethnic differences indicated the opposite trends, with Hispanic/Latinx veterans more likely to use telehealth specialty pain care than non-Hispanic/Latinx veterans during the on-set of the pandemic, even after adjusting for age, gender, and rurality. Further, disparities in use of any specialty pain care were exacerbated for several racial/ethnic minority groups at the start of COVID-19 and these differences were driven primarily by widening gaps in in-person pain care.

Increases in telehealth use across groups may point to enterprise-wide efforts to enhance VA’s capacity to deliver virtual care in response to COVID-19. Prior to COVID-19, VA had already expanded virtual care access by standardizing telehealth processes.^15^ Access was further enhanced during COVID-19, with VA directing all facilities to convert all in-person appointments to virtual care as clinically appropriate.^15^ Notably, VA tailored efforts to increase telehealth access to vulnerable and high-risk populations. These efforts are mirrored nationally: in one report, patients with Medicaid, Medicare, and private insurance had the higher rates of telehealth use during COVID-19 compared to uninsured patients.^38^ These findings may point to lack of insurance exacerbating the digital divide, and as such, disparities may be ameliorated in the VA as a universal healthcare system. Yet, we see from our study that telehealth use for pain care across individuals of color remained lower than White individuals during the start of the pandemic.

In-person specialty pain care disparities were larger at the start of COVID-19 compared to pre-COVID for Black, Asian, and NHOPI veterans. One potential explanation for this trend was that by the end of our study measurement period (i.e., November 2020), in-person specialty pain care was still limited. Simultaneously, telehealth specialty pain care grew across all subgroups, which may have mitigated reductions in in-person care.

Trends in which racial-ethnic minority veterans’ use of specialty pain care exceeded White veterans’ use were generally attenuated after adjusting for rurality, age, and gender. These trends speak to the importance of examining intersections of identity when measuring disparities as some between-group differences by race or ethnicity can be masked or magnified by other patient factors. One study found that when examining the intersections of age and race, Black and White individuals have similar levels of health-related technology use, but disparities emerge around age 62 and widen until age 76, remaining stable until old age.^42^ These findings are consistent with other studies documenting lower rates of health-related technology use among racial/ethnic minorities in older populations.^43^

### Limitations

Our study data are from veterans who are active users of VA healthcare, and our findings may not generalize to non-VA populations. Although we used best data practices to select and create our variables,^44^ our data are secondary administrative data. Thus, we are limited by the accuracy of the data captured in VA electronic health records. Additionally, while our analyses captured similar cohorts across two time periods, we were not able to examine outcomes longitudinally. We used a cross-sectional comparison approach, which limited our ability to account for specialty pain care patients may have received between their primary care visits and the assessment window. Finally, while our team used best practices to capture specialty pain care encounters,^44^ we must acknowledge limitations to using the 420 VA stop code. This code captures specialty pain care encounters across a wide range of services, including non-pharmacologic approaches (e.g., massage, acupuncture, psychological treatments, both primary care and pain clinic encounters). Although concerted efforts have been made by VA operations offices to standardize use of the 420 stop code across facilities, it may still be used inconsistently by some.

## CONCLUSION

While virtual care disparities in general were not exacerbated during the start of COVID-19, racial-ethnic disparities across pain care modalities persisted except for virtual care usage among individuals identifying as NHOPI and Hispanic/Latinx. Future research should investigate whether efforts to enhance pain care engagement among minoritized veterans and scaled telehealth expansion hold promise for reducing disparities and capitalize on health services engagement models (e.g., Andersen or Gelberg theoretical frameworks) to identify mechanisms of disparities.^45, 46^

### Supplementary Information

Below is the link to the electronic supplementary material.

Supplementary file1 (DOCX 33.6 KB)
