# Supplementary material for: Pain Care Disparities and the Use of Virtual Care Among Racial-Ethnic Minority Groups During COVID-19
Source: J Gen Intern Med. 2024 Jan 22;39(Suppl 1):68–78. doi: 10.1007/s11606-023-08473-0 (PMC10937888; doi:10.1007/s11606-023-08473-0)
Supplement: Supplementary file 1 — Supplementary file1 (DOCX 33.6 KB) [file 11606_2023_8473_MOESM1_ESM.docx]

| **Supplementary Appendix A. Standards for QUality Improvement Reporting Excellence (SQUIRE 2.0) Checklist** | | |
| --- | --- | --- |
| **SQUIRE Checklist Item** | **Description** | **Section/Page Number** |
| 1. Title | Pain Care Disparities and the Use of Virtual Care Among Racial-Ethnic Minority Groups During COVID-19 | Title Page |
| 1. Abstract | See Abstract text in manuscript | Abstract/pp. 2-3 |
| **Introduction** | | |
| 1. Problem Description | The COVID-19 pandemic caused an unprecedented disruption to healthcare operations and research, including specialty pain care. An abrupt pivot to virtual care caused concern that racial-ethnic minority individuals would bear the brunt of a digital divide. | Background/p. 4 |
| 1. Available Knowledge | See Background text in manuscript | Background/pp. 4-5 |
| 1. Rationale | Given historical injustices and the potential for disparities in virtual care access among veterans with chronic pain, it is of the utmost importance to identify whether disparities in virtual pain care exist in the post-COVID-19 world. | Background/p. 5 |
| 1. Specific Aims | The purpose of this study is to determine whether disparities in receipt of specialty pain care were exacerbated for racial-ethnic minority groups during the on-set of COVID-19, with a focus on virtual care. | Background/p. 5 |
| **Methods** | | |
| 1. Context | This was a retrospective national cohort study using administrative data derived from the VA Corporate Data Warehouse. All analytic cohort individuals were active users of VA primary care and had chronic pain. | Design and Participants/p. 6 |
| 1. Interventions | Specialty pain care, as described in the Background and Method | Background/p. 4 and Methods/p. 7 |
| 1. Study of the Interventions | We describe what comprises specialty pain care. These approaches include evidence-based, non-pharmacological approaches. | Background/p. 4 |
| 1. Measures | - Outcomes – Utilization of specialty pain care via telehealth, utilization of in-person specialty pain care, utilization of any specialty pain care - Independent measures – Racial and ethnic identity - Covariates – age, gender, and rurality | Main Measures/pp. 6-7 |
| 1. Analysis | Main analyses focused on descriptive differences between our two period cohorts and a quasi-Poisson regression modeling approach of receiving pain care during each period, adjusted for days of follow-up. We measured both unadjusted and adjusted models. | Analytic Approach, pp. 8-9 |
| 1. Ethical Considerations | All authors declare that they have no conflict of interest outside of funding sources for this work. This project was designated as quality improvement for the purposes of program implementation and evaluation. | Additional Information/p. 17 |
| **Results** | | |
| 1. Results | See Key Results section in manuscript for details | Key Results/pp. 9-12 |
| **Discussion** | | |
| 1. Summary | Key findings and takeaways are summarized in first paragraph of the Discussion | Discussion/p. 12 |
| 1. Interpretation | Our interpretation of the study’s findings can be found in the Discussion | Discussion/pp. 12-15 |
| 1. Limitations | See Limitations section of Discussion | Discussion/p. 14 |
| 1. Conclusions | See Conclusion section of Discussion | Discussion/p. 15 |
| **Other Information** | | |
| 1. Funding | This material is based upon work supported in part by the Department of Veterans Affairs, Veterans Health Administration, Office of Research and Development Small aWard Initiative For impacT (SWIFT; PI: Chen) and VA HSR&D Career Development Award IK2 HXX002866 (PI: Chen). | Acknowledgments/p. 16 |
| Source: Ogrinc G, Armstrong GE, Dolansky MA, Singh MK, Davies L. SQUIRE-EDU (Standards for QUality Improvement Reporting Excellence in Education): Publication Guidelines for Educational Improvement. Acad Med. 2019 Oct;94(10):1461-1470. PMID: 30998575 | | |

| **Supplementary Appendix B. VA Stop Codes Included and Excluded in Analyses** | |
| --- | --- |
| **Stop Code*** | **Definition** |
| 420 | Pain Management |
| 179 | Real Time Clinical Video Care |
| 690 | Real Time Clinical Video Telehealth Visit (originating patient station) |
| 692 | Real Time Clinical Video Telehealth Visit (provider station – same STA3) |
| 693 | Real Time Clinical Video Telehealth Visit (provider station – different STA3) |
| 723 | OEND^†^ Visit via Clinical Video Telehealth (originating patient site) |
| 724 | OEND Visit via Clinical Video Telehealth (provider site) |
| 683^‡^ | Home Telehealth Non-Video Monitoring |
| 684^‡^ | Home Telehealth Non-Video Intervention |
| 685^‡^ | Home Telehealth Program Patients |
| 719^‡^ | My Health*e*Vet Secure Messaging |
| *Notes.* *VA stop codes are 3-digit identifiers used to identify the workgroup responsible for outpatient encounters and inpatient services; ^†^OEND = Opioid Overdose Education and Naloxone Distribution; ^‡^VA stop code is excluded from our in-person pain care variable | |

| **Supplementary Appendix C. Characteristics of all VA patients with chronic pain stratified by receipt of in-person, telephone, or video specialty pain care encounter (row percentages represented)*** | | | | | | | | |
| --- | --- | --- | --- | --- | --- | --- | --- | --- |
| **Population characteristics stratified by type of specialty pain care encounter** | **Pre-COVID Cohort (n=1,536,954)** | | | | **COVID-era Cohort (n=1,649,053)** | | | |
|  | **Any encounter (n=100,065)** | **In-person**  **(n=96,533)** | **Telephone**  **(n=18,262)** | **Video**  **(n=1,897)** | **Any encounter (n=82,584)** | **In-person**  **(n=59,832)** | **Telephone**  **(n=38,837)** | **Video**  **(n=12,051)** |
|  | **N (%)** | **N (%)** | **N (%)** | **N (%)** | **N (%)** | **N (%)** | **N (%)** | **N (%)** |
| **Age, mean (SD)** | 58.18 (13.01) | 58.11 (13.02) | 59.30 (12.51) | 58.30 (12.96) | 58.49 (13.13) | 58.58 (13.13) | 59.10 (12.90) | 55.39 (13.22) |
| **Age (yrs)** |  |  |  |  |  |  |  |  |
| <30 | 2,002 (4.7) | 1,953 (4.6) | 241 (0.6) | 35 (0.08) | 1,556 (3.5) | 1,085 (2.4) | 650 (1.5) | 325 (0.7) |
| 30-39 | 8,567 (6.8) | 8,319 (6.6) | 1,330 (1.1) | 174 (0.1) | 7,172 (5.1) | 5,128 (3.6) | 3,094 (2.2) | 1,451 (1.0) |
| 40-49 | 13,946 (8.3) | 13,525 (8.1) | 2,284 (1.4) | 243 (0.1) | 11,377 (6.3) | 8,318 (4.6) | 4,991 (2.7) | 2,036 (1.1) |
| 50-59 | 24,623 (8.5) | 23,791 (8.2) | 4,430 (1.5) | 461 (0.2) | 19,740 (6.5) | 14,248 (4.7) | 9,154 (3.0) | 3,210 (3.0) |
| 60-69 | 32,131 (6.6) | 30,914 (6.3) | 6,269 (1.3) | 623 (0.1) | 25,004 (5.3) | 18,057 (3.8) | 12,269 (2.6) | 3,223 (0.7) |
| 70-79 | 15,486 (5.2) | 14,876 (5.0) | 3,055 (1.0) | 297 (0.1) | 15,010 (4.0) | 10,966 (2.9) | 7,324 (1.9) | 1,596 (0.4) |
| 80-89 | 3,012 (3.0) | 2,866 (2.8) | 607 (0.6) | 60 (0.06) | 2,508 (2.3) | 1,867 (1.7) | 1,254 (1.2) | 191 (0.2) |
| 90+ | 297 (1.5) | 288 (1.5) | 46 (0.2) | 4 (0.02) | 216 (1.1) | 162 (0.8) | 101 (0.5) | 19 (0.09) |
| **Race** |  |  |  |  |  |  |  |  |
| White | 70,516 (6.6) | 67,796 (6.3) | 13,596 (1.3) | 1,532 (0.1) | 58,253 (5.1) | 42,371 (3.7) | 27,810 (2.4) | 8,203 (0.7) |
| Black | 19,893 (6.3) | 19,408 (6.1) | 3,017 (1.0) | 210 (0.07) | 16,064 (4.7) | 11,550 (3.4) | 7,169 (2.1) | 2,531 (0.7) |
| Asian | 990 (6.5) | 975 (6.4) | 130 (0.9) | 4 (0.03) | 836 (4.7) | 621 (3.5) | 326 (1.8) | 167 (0.9) |
| NHOPI^†^ | 930 (6.5) | 905 (6.3) | 129 (0.9) | 14 (0.1) | 809 (5.1) | 543 (3.4) | 404 (2.6) | 134 (0.9) |
| AI/AN^‡^ | 867 (6.8) | 834 (6.5) | 163 (1.3) | 20 (0.2) | 682 (4.9) | 508 (3.6) | 310 (2.2) | 96 (0.7) |
| 2+ races | 1,048 (7.0) | 1,012 (6.7) | 174 (1.2) | 19 (0.1) | 859 (5.2) | 605 (3.7) | 421 (2.6) | 126 (0.8) |
| Unknown/missing | 5,821 (6.3) | 5,603 (6.1) | 1,053 (1.1) | 98 (0.1) | 5,081 (5.0) | 3,634 (3.6) | 2,397 (2.4) | 794 (0.8) |
| **Ethnicity** |  |  |  |  |  |  |  |  |
| Hispanic/Latinx | 7,495 (7.1) | 7,338 (7.0) | 910 (0.9) | 79 (0.07) | 5,912 (5.0) | 3,967 (3.4) | 3,006 (2.6) | 1,067 (0.9) |
| Non-Hispanic/Latinx | 85,942 (6.4) | 82,812 (6.2) | 16,078 (1.2) | 1,721 (0.1) | 70,860 (5.0) | 51,706 (3.6) | 33,116 (2.3) | 10,117 (0.7) |
| Unknown/missing | 6,628 (7.0) | 6,383 (6.7) | 1,274 (1.3) | 97 (0.1) | 5,812 (5.5) | 4,159 (3.9) | 2,715 (2.6) | 867 (0.8) |
| **Gender** |  |  |  |  |  |  |  |  |
| Women | 13,964 (8.4) | 13,553 (8.1) | 2,214 (1.3) | 244 (0.1) | 11,822 (6.3) | 8,517 (4.6) | 5,197 (2.8) | 2,266 (1.2) |
| **Rural/urban dwelling** |  |  |  |  |  |  |  |  |
| Urban | 68,578 (7.0) | 66,400 (6.8) | 12,123 (1.2) | 990 (0.1) | 56,038 (5.3) | 40,129 (3.8) | 26,590 (2.5) | 8,867 (0.8) |
| Rural | 27,975 (5.9) | 26,827 (5.6) | 5,484 (1.1) | 763 (0.2) | 23,651 (4.6) | 17,558 (3.4) | 10,836 (2.1) | 2,875 (0.6) |
| Highly rural | 3,455 (5.3) | 2,966 (4.9) | 584 (1.0) | 134 (0.2) | 2,540 (4.0) | 1,925 (3.1) | 1,202 (1.9) | 240 (0.4) |
| Insular island | 15 (1.2) | 13 (1.0) | 3 (0.2) | 0 (0.0) | 50 (3.4) | 21 (1.4) | 43 (2.9) | 24 (1.6) |
| **Marital status** |  |  |  |  |  |  |  |  |
| Divorced/separated | 30,114 (7.0) | 36,614 (8.6) | 5,624 (1.3) | 547 (0.1) | 24,668 (5.5) | 22,357 (5.0) | 11,816 (2.6) | 3,555 (0.8) |
| Married or cohabiting | 53,969 (6.4) | 52,054 (6.2) | 9,835 (1.2) | 1,111 (0.1) | 44,818 (4.9) | 32,719 (3.6) | 20,879 (2.3) | 6,587 (0.7) |
| Never married/single | 11,688 (6.6) | 11,323 (6.4) | 1,989 (1.1) | 164 (0.09) | 9,640 (4.6) | 6,947 (3.6) | 4,487 (2.3) | 1,505 (0.8) |
| Widowed | 3,500 (4.8) | 3,356 (4.6) | 689 (0.9) | 65 (0.09) | 2,758 (3.8) | 1,986 (2.7) | 1,334 (1.8) | 317 (0.4) |
| Unknown | 794 (5.5) | 774 (5.4) | 125 (0.9) | 10 (0.07) | 700 (4.0) | 497 (2.8) | 321 (1.8) | 45 (0.5) |
| **Pain diagnostic category** |  |  |  |  |  |  |  |  |
| Fibromyalgia | 5,113 (17.1) | 4,991 (16.7) | 847 (2.8) | 88 (0.3) | 3,536 (12.9) | 2,583 (9.4) | 1,689 (6.2) | 674 (2.5) |
| Neck pain | 20,450 (12.9) | 19,805 (12.5) | 3,837 (2.4) | 361 (0.2) | 18,369 (9.9) | 13,467 (7.2) | 8,872 (4.8) | 2,734 (1.5) |
| Orofacial, ear, & temporomandibular disorder pain | 554 (12.8) | 537 (12.4) | 107 (2.5) | 8 (0.2) | 514 (10.3) | 400 (8.0) | 237 (4.7) | 77 (1.5) |
| Other painful conditions^§^ | 23,344 (12.1) | 22,429 (11.7) | 4,670 (2.4) | 575 (0.3) | 20,833 (9.7) | 14,738 (6.9) | 10,506 (4.9) | 3,551 (1.7) |
| Back pain | 64,397 (10.9) | 62,177 (10.5) | 12,739 (2.2) | 1,083 (0.2) | 54,941 (8.5) | 39,905 (6.2) | 26,771 (4.1) | 8,054 (1.2) |
| Headache | 7,585 (10.8) | 7,406 (10.6) | 1,148 (1.6) | 111 (0.2) | 6,869 (8.2) | 5,165 (6.2) | 2,944 (3.5) | 1,227 (1.5) |
| Limb/extremity pain, joint pain, etc.^¶^ | 55,234 (6.5) | 53,436 (6.3) | 9,859 (1.2) | 981 (0.1) | 46,639 (5.0) | 34,114 (3.7) | 21,896 (2.4) | 6,908 (0.7) |
| Urogenital, pelvic, & menstrual pain | 1,068 (8.6) | 1,035 (7.4) | 184 (1.3) | 17 (0.1) | 999 (6.4) | 722 (5.6) | 470 (3.0) | 171 (1.1) |
| Fractures, contusions, sprains, & strains | 3,467 (7.4) | 3,366 (7.2) | 635 (1.4) | 62 (0.1) | 3,052 (5.9) | 2,239 (4.3) | 1,434 (2.8) | 445 (0.9) |
| Musculoskeletal chest pain | 3,848 (7.0) | 3,724 (6.7) | 671 (1.2) | 62 (0.1) | 3,301 (5.6) | 2,451 (4.2) | 1,541 (2.6) | 463 (0.8) |
| Abdominal & bowel pain | 6,989 (6.4) | 6,755 (6.2) | 1,274 (1.2) | 131 (0.1) | 6,287 (5.2) | 4,593 (3.8) | 2,946 (2.5) | 924 (0.8) |
| Systemic disorders or diseases causing pain | 2,444 (6.3) | 2,342 (6.0) | 462 (1.2) | 77 (0.2) | 2,229 (5.3) | 1,586 (3.8) | 1,072 (2.6) | 357 (0.9) |
| Neuropathy | 12,072 (5.7) | 11,551 (5.5) | 2,353 (1.1) | 286 (0.1) | 10,318 (4.5) | 7,453 (3.3) | 5,077 (2.2) | 1,423 (0.6) |

| *Notes. **For example, the percentage reflected for individuals <30 years old is calculated as [(2,002/42,290) x 100] which is the percentage of Veterans under 30 years old in the pre-COVID era that received any specialty pain care. The denominator, 42,290, comes from Supplementary Appendix Table A (i.e., the total number of Veterans under the age of 30 in the pre-COVID era cohort). Note also that types of encounters were not mutually exclusive, i.e., an individual in the study cohort could have received both in-person & telephone care in the 6-month follow-up period; †NHOPI = Native Hawaiian or Other Pacific Islander; ^‡^AI/AN = American Indian or Alaska Native; ^§^Other painful conditions include sickle cell disease, Complex Regional Pain Syndrome, systemic lupus erythematosus, acquired deformities, spinal cord injury, Lyme Disease ^¶^Limb/extremity pain, joint pain, & non-systemic, non-inflammatory arthritic disorders |
| --- |
